# Emotional conflict adaptation predicts intrusive memories

**DOI:** 10.1371/journal.pone.0225573

**Published:** 2020-02-20

**Authors:** Marcus Grueschow, Iva Jelezarova, Maren Westphal, Ulrike Ehlert, Birgit Kleim

**Affiliations:** 1 Zurich Center for Neuroeconomics (ZNE), Department of Economics, University of Zurich, Zurich, Switzerland; 2 Experimental Psychopathology and Psychotherapy, Dept. of Psychology, University of Zurich, Zurich, Switzerland; 3 Department of Psychiatry, Psychotherapy and Psychosomatics, University Hospital of Psychiatry, University of Zurich, Zurich, Switzerland; 4 Department of Psychology, Pace University, Pleasantville, United States of America; 5 New York State Psychiatic Institute, Columbia University, New York, United States of America; 6 Clinical Psychology and Psychotherapy, Dept. of Psychology, University of Zurich, Zurich, Switzerland; Victoria University of Wellington, NEW ZEALAND

## Abstract

Why do some individuals experience intrusive emotional memories following stressful or traumatic events whereas others do not? Attentional control may contribute to the development of such memories by shielding attention to ongoing tasks from affective reactions to task-irrelevant emotional stimuli. The present study investigated whether individual differences in theability to exert cognitive control are associated with experiencing intrusive emotional memories after laboratory trauma. Sixty-one healthy women provided self-reported and experimentally derived measures of attentional control. They then viewed a trauma film in the laboratory and recorded intrusive memories for one week using a diary. Gaze avoidance during trauma film exposure was associated with more intrusive memories. Greater attentional control over emotion prior to film viewing, as assessed with the experimental task, predicted fewer intrusive memories while self-reported attentional control was unrelated to intrusive memories. Preexisting capacity to shield information processing from distraction may protect individuals from developing intrusive emotional memories following exposure to stress or trauma. These findings provide important clues for prevention and intervention science.

## Introduction

Unbidden experience of emotional memories is a core symptom of posttraumatic stress disorder (PTSD) and other trauma and stressor-related disorders [1]. They typically consist of sensory impressions, such as visual, auditory or bodily sensations, and emotional responses to the trauma that come to mind involuntarily and can be triggered by internal or external stimuli, often outside of one’s awareness [2–4]. Intrusive emotional memories, including both undesirable thoughts and images, are a transdiagnostic phenomenon and thus hold relevance for the understanding and treatment of psychopathology beyond PTSD [5, 6]. Despite a large body of research on potential risk and protective factors in trauma-related disorders such as PTSD [7–9], comprehensive predictors and mechanisms that predispose individuals to develop intrusive emotional memories are still not fully understood [4, 10] (see also [11] for a comprehensive review of pretrauma-predictors of PTSD).

Cognitive control comprises a number of candidate processes, such as response inhibition, conflict monitoring, decision making and cognitive flexibility, that may all be involved in the development of intrusive emotional memories [12–14]. These mental operations that jointly allow variation in information processing and behavior to meet contextual demands. They include maintaining a current goal in working memory and the ability to resolve conflict between task-relevant stimuli and distractors [15, 16]. This conflict resolution ability likely is critical to effective emotion regulation and goal-oriented behavior in trauma survivors facing trauma-related and other emotionally salient cues. Pursuing trauma-unrelated tasks requires attentional resources to be tuned to task-relevant activities in the presence of strong negative emotions that may trigger intrusive memories.

Accordingly, individual differences in these cognitive control functions may be implicated in the development of intrusive emotional memories. Consistent with this idea, previous studies have found that deficits in the ability to resist proactive interference are associated with increased vulnerability to intrusive emotional memories [17–19]. The latter study found cognitive control to predict intrusion frequency, but this finding did not hold for intrusion distress and vividness once depression and emotional arousal were controlled for. Inhibitory control is a related construct that has been associated with intrusions in clinical populations as well as in the laboratory [20, 21]. Greater PTSD symptom severity was also associated with more time spent looking at background contexts and less time looking at target faces, which is consistent with greater susceptibility to distraction by task-irrelevant emotional stimuli [22]. Finally, James et al., (2016) investigated whether playing Tetris, i.e. engaging in a working memory task, prior to trauma film viewing did not interfere with intrusive memory development, as no differences between experimental and control groups emerged. Together, these findings point to a possible role of cognitive control, but results are mixed (see also [10], for a review) and some studies are solely based on self-report measures of attention and cognitive control. Self-report surveys may be undermined by factors, such as high distress, emotional arousal and limited introspective ability. Experimental measures are independent of individuals’ explicit self-reports and allow for testing a theoretically well-grounded behavioral measure (conflict adaptation) for predicting the amount of intrusive memories. Crucially, no study so far has indexed the predictive value of the capacity for cognitive control in the context of emotional material and particularly memory intrusions.

The present study extends previous findings by investigating whether an enhanced cognitive control ability in the face of emotional distraction reduces the likelihood of experiencing intrusive emotional memories after exposure to distressing stimuli. Selective attention paradigms such as the classic Stroop task capture cognitive control processes that mediate conflict resolution [23]. Egner and colleagues modified the Stroop task to examine emotional conflict [24, 25] by presenting images of happy or fearful faces that are overlaid with the words "happy" or "fear". The task is to identify the expression of the face as fearful or happy and to ignore the overlaid word. Incongruent stimuli (e.g., a fearful face with overlaid word "happy") create emotional conflict, resulting in longer reaction times. This slowdown in reaction time is lessened, however, if the previous image was also incongruent. A possible explanation of this improvement in conflict resolution is that conflict in an incongruent trial may upregulate selective attention, which may carry-over onto the next trial [26, 27]. In this context, faster reaction time serves as an index of conflict adaptation, which reflects the ability to adapt emotional processing from stimulus to stimulus. Deficits in emotion regulation and related regulatory processes have been linked to the etiology, development, and maintenance of diverse psychological symptoms [28] and could thus be a transdiagnostic feature. Such processes include experiential avoidance, suppression and context insensitivity, which may all potentially be governed by mechanisms of cognitive control. A behavioral index of cognitive control, such as a conflict adaptation score may thus provide a potentially useful tool to identify a transdiagnostic risk factor for psychological disorders [28]. In fact, this index has previously been successfully exploited in showing that individuals with generalized anxiety disorder exhibit decreased conflict adaptation scores [29]. This study, however, did not examine whether conflict adaptation deficits prospectively predict vulnerability to psychopathology.

The primary goal of the current study was to examine whether individual differences in cognitive control, indexed by conflict adaptation, prospectively predicts the development of intrusive emotional memories in healthy individuals exposed to trauma-related stimuli. We aim to index individual differences using a conflict adaptation score, an established behavioral measure of cognitive control. Crucially, we acquire this score from our subjects prior to viewing an adverse trauma film, which may potentially induce intrusive memories. We hypothesised that low conflict adaptation ability would predict greater frequency of intrusive memories following exposure to traumatic content in the laboratory. We also explored whether conflict adaptation as an implicit measure of attentional control would be related to a self-report explicit measure of attentional control.

## Materials and methods

### Participants and procedure

Participants were recruited through student mailing lists and online advertisements. Sixty-one healthy female volunteers between the ages of 18 and 35 years (mean age 25.11 years, *SD = 5*.*32*) were included in the present study and compensated with either 50 CHF (56 USD) or three credit points. Expecting a small to medium effect size for the predictive power of conflict adaptation, a sample size of 35 is sufficient to detect the effect, given a power of .80 and a two-tailed significance level of .05. A telephone screening was conducted in order to screen for current psychiatric illnesses, and previous direct or indirect experience of interpersonal violence. Screening questions were derived from standardized questionnaires (Structure Clinical Interview for Psychiatric Diagnoses, SCID: question on previous depression), as well as formulated specifically for this study (exposure to interpersonal violence). The local ethic board at the University of Zurich approved the study.

The study was conducted during the hours of 11 am and 3pm. This time range was chosen to control for potential circadian effects, as well as effects of sleep after trauma exposure, which we have indeed observed in an earlier study using the same trauma film paradigm. In the beginning of the experimental session participants were informed about the potentially traumatic film content. After providing informed consent, they were asked to complete a set of demographic and clinical questionnaires (including the instruments described above), which took approximately 30–45 minutes. They then completed the emotional conflict task and were shown the trauma film while the experimenter left the room. At the end of the experimental session, participants were instructed how to keep the intrusion diary and a date and time for the follow-up session was arranged.

In the follow-up session, the experimenter reviewed the returned intrusion diary while participants filled out a short questionnaire. Participants were debriefed and received compensation.

### Self-report questionnaires

#### Demographic information

Participants’ age, nationality, marital status, employment, use of psychotropic drugs and consumption of nicotine, cannabis and alcohol was assessed.

#### Depressive symptoms

Symptoms of depression were assessed with the Beck Depression Inventory (BDI; German version by [30]). The BDI consists of 21 self-report items ranging between zero and three, which are being added up to create a sum score. The BDI sum score ranges between zero and 63, whereby high scores indicate more severe symptoms of depression. The BDI has shown high internal consistency, Cronbach’s α = .73 to α = .92. In this sample the BDI Cronbach’s α was .78.

#### Previous Trauma

Previous exposure to traumatic life events were assessed with a shortened and modified version of the Post-Traumatic Stress Diagnostic Scale by Foa et al. [31]. The 16 items inquired if the participants had previously experienced different stressful life events, such as severe illness, death of a family member or being unemployed. If they had experienced some of the prompted incidents, they were asked to indicate the degree of subjective stressfulness on a scale ranging from zero ("not stressful at all") to three ("extremely stressful").

### Self-report attentional control

Self-reported attentional control was measured with a German version of the Attentional Control Scale (ACS) by Derryberry and Reed[32]. 20 items ranging from one ("almost never") to four ("always") assess the ability to focus on a task while being exposed do multiple stimuli (e.g. "I can concentrate well even if there is music in the next room") or the ability to shift between different tasks (e.g. I can easily write or read while speaking on the phone"). Half of the items need to be reversed for the determination of the ACS sum score. Low scores indicate low attentional control, whereas high scores indicate more effective attentional control. The ACS has shown high internal consistency scores (Cronbach’s α = .88;[32]). Cronbach’s α in the present sample was high, α = .82.

### Experimental attentional control

**Fig 1:**
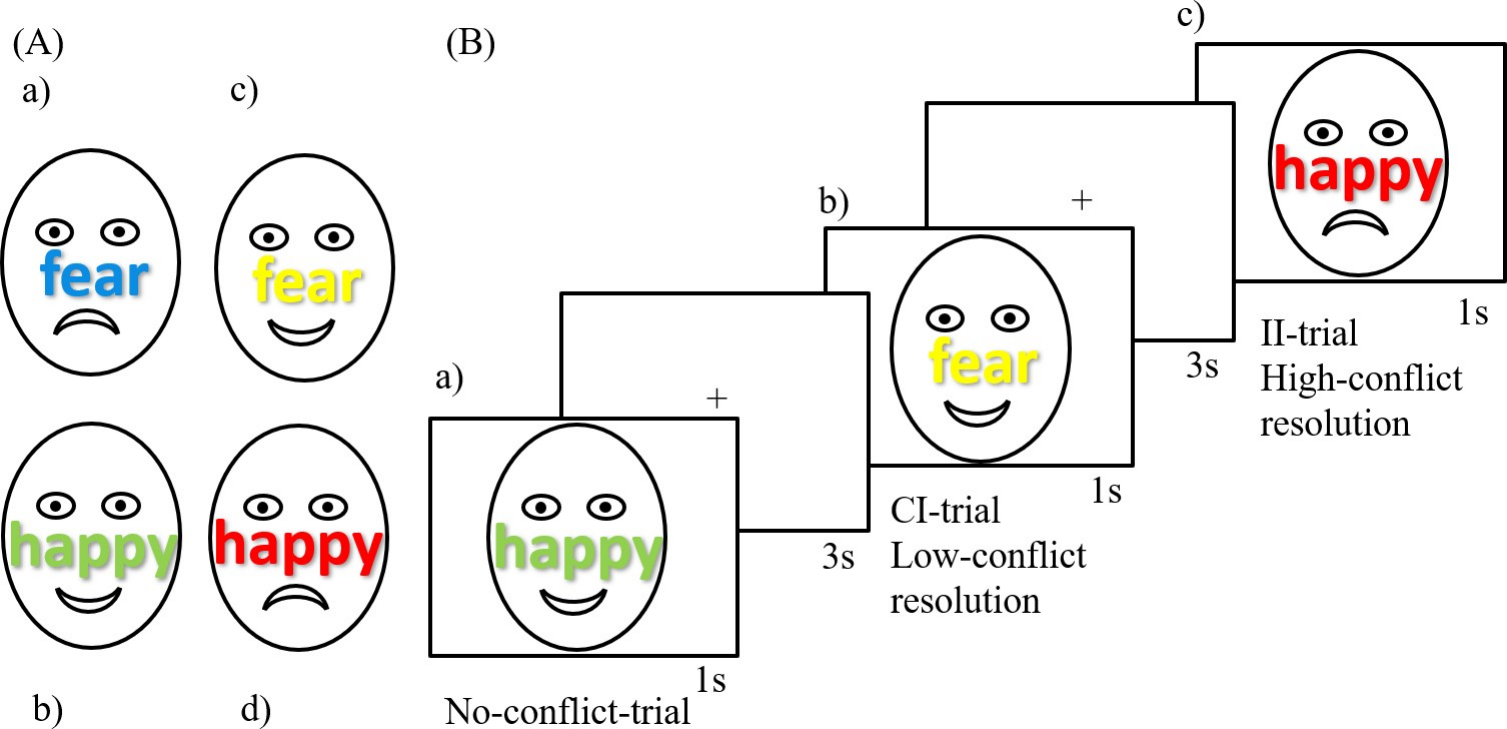
Exemplary stimuli of the ECT conditions used in the present study. Face stimuli used in our experiment were identical to the face stimuli used in Etkin et al. 2006 and comprised of face pictures of real people. (A) a) and b) show congruent conditions, c) and d) incongruent conditions. Participants were instructed to react to the facial expression only and to ignore the overlaid word and to answer as fast and as accurate as possible. Stimuli were displayed for 1s with intermediate intervals of 3s (B) a) shows a congruent condition, b) an incongruent condition preceded by a congruent one (CI) and c) shows an incongruent condition preceded by an incongruent one (II).

A modified emotional Stroop task was used to index conflict adaptation [15, 25]. As shown in Fig 1, each trial included (1) the presentation of a central fixation cross for 1000ms, (2) the presentation of an emotional face [33] (i.e., happy or fearful) with an overlaid emotional word (i.e., happy or fear). Participants were asked to indicate whether the facial expression of the stimulus was happy or fearful by pressing the corresponding arrow on the laptop keyboard (left for happy, right for fearful) and to disregard the emotional word and to respond as fast and accurate as possible. A total of 120 trials were presented (60 congruent, 60 incongruent), with a duration of approximately 14 minutes. The task was presented on a 9.45 x 14.17 square inch laptop using Cogent 2000 (http://visilab.ucl.ac.uk/cogent_2000.php) running in MATLAB (MathWorks, Natick, MA, USA).

As presented in Fig 1, stimulus conditions could either be congruent (C), e.g., happy face and word "happy", or incongruent (I), e.g., happy face and word "fear". Trials can furthermore be classified into high and low conflict resolution trials. High conflict resolution trials are defined as incongruent trials preceded by another incongruent trial (II), while low conflict resolution trials are incongruent trials preceded by congruent trials (CI). It is assumed that during incongruent trials an upregulation mechanism mobilizes cognitive resources to meet the current task demands (in this case resolving emotional conflict). This mechanism is believed to involve the noradrenergic arousal system [34]. Conflict resolution on a subsequent incongruent trial will benefit from this arousal effect, as much less upregulation is needed because of a carry-over from the previous trial. The behavioral consequence is reduced RTs on II trials as compared to CI trials which do not benefit from any previous trial upregulation of cognitive resources because there was no conflict on the previous trial adaptation [15, 16, 25, 27, 35].

Following Etkin et al. (2006) and Monti et al. (2010), individual conflict adaptation scores were calculated from reaction times: using the following formula: conflict adaptation (CA) = [(CI-CC)-(II-IC)]. This conflict adaptation score indexes the interaction between previous and current trial on reaction times with higher values reflecting reduced conflict (higher conflict resolution) following incongruent as compared to congruent trials, i.e., enhanced conflict (low conflict resolution). We hypothesized that individuals with higher conflict adaptation i.e: enhanced cognitive control are capable of better shielding their attention during analog trauma viewing which consequently results in less intrusive memories. In addition to computing individual CA-scores, we also calculated a dichotomous variable with two groups reflecting increased conflict adaptation (CA-score > 0) and reduced conflict adaptation (CA-score ≤ 0).

### Trauma film

Participants were shown a continuous film sequence from the R-rated French motion picture “Irréversible” by Gaspar Noë (2002). The clip was approximately 12 minutes long and showed a young woman walking through an underpass and then being brutally raped. The same film sequence has been shown to induce analogue symptoms of post-traumatic stress disorder in healthy participants, such as intrusive memories [36]. The clip was displayed on a 9.45 x 14.17 square inch laptop screen in a dark room. Participants were given the instruction to imagine being a close witness of the incident.

In order to examine the immediate effects of the trauma film, participants were asked to rate their affect and arousal before and after the film using the Self-Assessment Manikin Scale [37] on a valence scale from 0 to 9 (higher scores indicate negative affect and greater arousal, respectively). As expected, participants indicated significantly more negative affect following the trauma film, *t* = -9.31, *p* < .001 (*M*_pre_ = 2.56, *SD*_pre_ = 1.09, *M*_post_ = 4.95, *SD*_post_ = 1.84) and significantly more arousal, t = -9.81, p < .001 (*M*_pre_ = 2.51, *SD*_pre_ = 1.31, *M*_post_ = 5.22, *SD*_post_ = 1.93).

### Gaze avoidance

Participants were asked to indicate the degree to which they deliberately turned their eyes away from the screen on a 5-point scale ranging from 1 (“not at all”) to 5 (“very frequently”).

### Intrusion diary

Participants were asked to keep a diary for seven days following viewing the film and to record all intrusive memories related to the trauma film. For each recorded memory, they specified the time of occurrence, the content of the memory, and whether it was an intrusive image, thought or both. On a scale from 1 to 10 they indicated how vivid and how stressful they experienced the memory and whether the memory occurred involuntarily or not. Upon returning the diaries, all recordings were reviewed by the examiner. If necessary, participants were asked to clarify or provide further information regarding the intrusiveness of the recorded memories.

### Analysis

Data was analyzed using SPSS (IBM SPSS Statistics, version 24). In accord with Monti et al. (2010), we removed reaction time outlier-trials that deviated more than 1 standard deviation from individual means, leading to 0.01% of trials being excluded from the data. Zero-order correlations were computed using Pearson’s coefficient. An ANOVA was used to calculate differences in total intrusion count between individuals with increased versus reduced conflict adaptation scores. Prediction of intrusion frequency with attentional and emotional control was analyzed using a hierarchical linear regression, including depression, prior trauma exposure and gaze avoidance as covariates.

## Results

**Fig 2:**
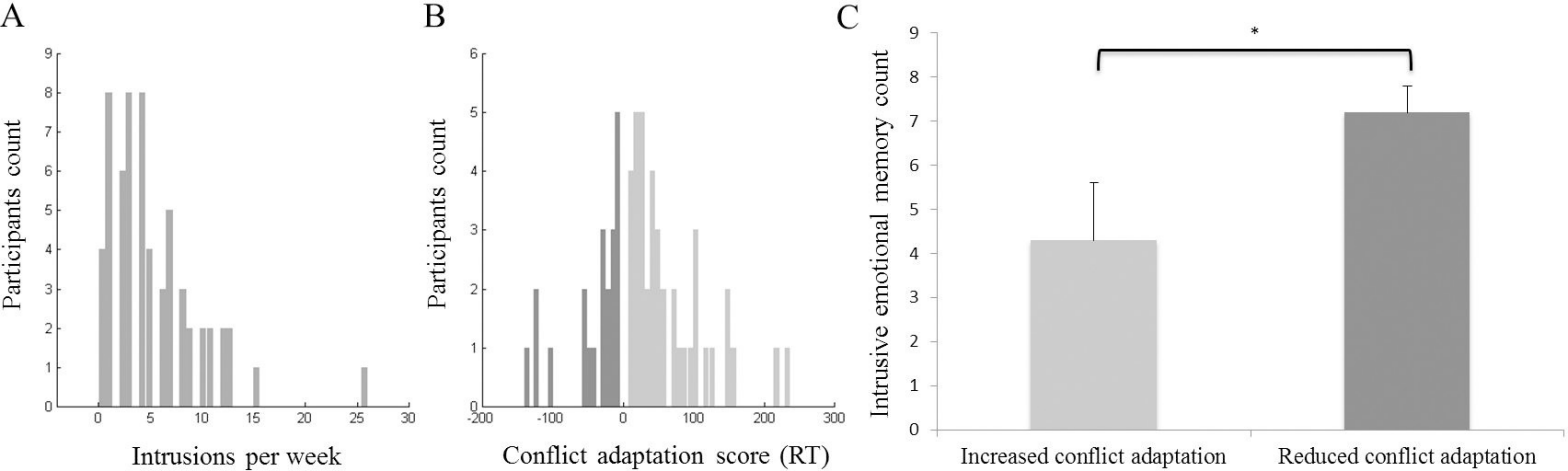
Intrusion frequency and conflict adaptation score (*N* = 59). Range of intrusion frequencies during the week of keeping the diary (A), range of conflict adaptation scores, with reduced conflict adaptation in dark and increased conflict adaptation depicted as light bars (B), and differences between individuals with increased (light bar) versus reduced (dark bar) conflict adaptation in intrusive emotional memory count, in number intrusion experienced during the week of conducting the diary (C). Note: * p< .05, B and C: CA scores below zero (reduced conflict adaptation, light bar) and above zero (increased conflict adaptation, dark bar).

Zero-order correlations as well as means and standard deviations are shown in Table 1. On average, participants reported *M* = 5.31, *SE* = 4.63 intrusive memories during the week following the trauma film (range 0–26), Fig 2A, consisting mostly of pictures, 41%, followed by a mixture of pictures and thoughts, 34%, and intrusive thoughts, 25%. We replicated previous findings and found a significant conflict adaptation effect (t58 = 2.99, p = 0.004, t-test on CA > 0, two-tailed,) indicating that conflict on incongruent trials is resolved faster when preceded by incongruent trials compared to when preceded by congruent trials, which do not entail the resolution of conflict and related up-regulatory processes *[34]*. The mean conflict adaptation effect was *M* = 31.4 ms, *SD* = 78.3 ms. There were inter-individual differences in conflict adaptation scores, ranging from -140 to 237 ms, with 21 individuals (35.6%) with CA score in the negative range indicating reduced conflict adaptation, see Fig 2B. Conflict adaptation scores were unrelated to self-reported attentional control (r = 0.08, p<0.05).

**Table 1:** Zero-order correlations (N = 59).

	*Mean*	*SD*	1	2	3	4	5	6
1. Intrusion Frequency	5.31	4.63	-					
2. Attentional control (ACS)	54.56	7.96	.12	-				
3. Attentional control under emotion (ECT)	31.37	78.32	-.29*	.08	-			
4. Depression (BDI)	4.85	4.32	.16	-.15	-.10	-		
5. Number of previous trauma	2.81	1.81	-.14	-.05	-.04	.25*	-	
6. Gaze avoidance	1.84	1.13	.31*	-.05	-.06	-.04	.20	-

Note

* p< .05, ECT = Emotional Conflict Task, reaction time in ms, ACS = Attentional Control Scale, BDI = Beck Depression Inventory, gaze avoidance was indexed on a scale from 0 to 5, with 5 indicating very frequent gaze avoidance

As expected and displayed in Fig 2(C), those with conflict adaptation scores above zero (*n* = 39, 66%) experienced significantly fewer intrusive memories compared to those with reduced conflict adaptation scores, i.e. scores below zero (*n* = 20, 34%), *F* (1, 58) = 5.67, *p* = .021, Cohen's *d* = .61

Table 2 presents results of the hierarchical regression analysis on number of intrusive memories. Depression, prior trauma and gaze avoidance were included in a first step of the regression and explained 17% of the variance. The effect was driven by gaze avoidance, which was positively associated with the number of intrusive memories, indicating that those who did not maintain attention towards the movie throughout the experiment and turned away from the film experienced more intrusive memories later. Attentional control, as indexed by self-report and introduced in a second step, did not significantly predict intrusive memories over and above depression, prior trauma exposure and gaze avoidance. Cognitive control, as indexed by the conflict adaptation score and included in the third step, significantly increased the explained variance in intrusion frequency to a total of 25%, Cohen's *f*^*2*^ = .11. To test if our results are robust to extreme data points, we re-analyze the regression between intrusions per week and conflict adaptation score excluding outliers at upper and lower ends of the distribution in the dependent variable and find that the relationship is even more statistically significant (R = -0.3184, p = 0.0168), with normally distributed residuals (p = 0.1237, Lilliefors-test). This finding also holds for non-parametric statistics (Spearman’s Rho = -0.3469, p = 0.008) and when controlling for self-reported attentional control, depression, prior trauma exposure and gaze avoidance (multiple regression, t_50_ = -2.33 p = 0.024), further supporting our conclusions. Individuals with greater emotional control (and greater conflict adaptation scores) experienced fewer intrusive memories in the week post-exposure to the trauma-film.

**Table 2:** Attentional control over emotion predicts intrusions over and above self-reported attentional control and control variables (N = 59).

	*R^2^*	*p*	*ΔR*^2^	*B*	*SE(B)*	*β*
Step 1^a^	.15*	.029	.15*			
Depression (BDI)				.22	.14	.20
Number of previous trauma				-.33	.34	-.13
Gaze avoidance				1.23	.54	.30*
Step 2^b^	.17*	.033	.024			
Depression (BDI)				.24	.13	.23
Number of previous trauma				-.33	.33	-.13
Gaze avoidance				1.23	.53	.30
Self-reported Attentional Control (ACS)				.09	.15	.16
Step 3^c^	.25**	.009	.072**			
Depressiveness (BDI)				.23	.13	.22
Number of previous trauma				-.35	.32	-.14
Gaze avoidance				1.13	.52	.27*
Self-reported Attentional Control (ACS)				1.00	.07	.17
Affective control (ECT)				-.02	.01	-.27*

*Note*. Outcome variable = number of intrusions recorded in the diary during one week after exposure to trauma film

*p < .05

**p < .01; BDI = Beck Depression Inventory; ACS = Attentional Control Scale, ECT = Emotional Conflict Task

^a^*R* = .39, *F*(3, 55) = 3.23, *p* = .029.

^b^*R* = .42, *F*(4, 54) = 2.84, *p* = .033.

^c^*R* = .50, *F*(5, 53) = 3.44, *p* < .001

## Discussion

Enhanced cognitive control was associated with reduced intrusive emotional memories following a laboratory stressor. As expected, individuals with greater capacity to cognitively control emotional distraction (indexed by faster reaction times to incongruent trials preceded by incongruent trials) reported fewer intrusive memories over the course of one week after experimental trauma exposure. These findings are consistent with previous experimental research relating cognitive control to intrusive memories, which found that resistance to proactive interference was associated with fewer intrusive trauma memories [19, 38] and reduced working memory capacity was associated with increased PTSD symptoms. The present study extends this literature by investigating whether individual differences in cognitive control predicts subsequent intrusive re-experiencing of distressing film scenes. The finding that the experimental index of cognitive control was independent of attentional control assessed via self-report is consistent with research suggesting that explicit and implicit measures of attentional control may target different constructs [39].

Our data revealed the interesting observation that self-reported measures of attentional control did not relate to intrusive memories while a behavioral conflict adaptation score did. We believe the reason for this mismatch is that the behavioral score is a manifestation of neurobiological properties of an individuals’ cognitive control system that makes her eventually more or less susceptible to intrusive memories while self-reports may be driven by a number of biases which could have the potential to distort the quality, validity and reproducibility of the respective index. These biases may include individual differences in honesty and management of self-image appearance, introspective abilities, understanding and comprehension of the particular concept at question, response bias, as well as differences in interpretation and meaning of the scale points in rating scales. In addition, almost all self-report measures produce ordinal data, which only allows for rank ordering, and does not consider the distances between the obtained units. In contrast, our conflict adaptation score produces interval data, the units of which can directly be put in relation to one another and are hence much more interpretable.

Cognitive control ability predicted intrusion frequency over and above depression, prior trauma and gaze avoidance also expands previous research and suggests that deficits in cognitive control may play a unique role in predicting intrusions. This is also in line with conclusions of a recent review on predictors of intrusions that data-driven aspects of the event, rather than chronological or meaning-based aspects experienced during the event leads to intrusive re-experiencing [10] In fact, attentional control deficits could lead to increased data-driven processing and thus development of intrusive memories and PTSD.

Interestingly, greater gaze avoidance during trauma film exposure was associated with more intrusive memories. It is conceivable that turning away from trauma stimuli resulted in more fragmented trauma memories and thus more intrusive memories, despite actual decrease in viewing time and stimuli exposure. The observed positive relationship between gaze avoidance and intrusive memories may reflect a consequence of reduced cognitive control and may have additionally resulted in reduced memory encoding. More specifically, individuals with reduced conflict adaptation may revert to gaze avoidance as a last resort if an individuals’ cognitive control mechanisms cannot appropriately shield against adverse stimuli anymore. It follows, that individuals with reduced conflict adaptation should exhibit enhanced intrusive memories, which is exactly what we observed in our data. Moreover, gaze avoidance may additionally result in reduced encoding. It has previously been reported that reduced encoding leads to fragmented memories, which in turn has been shown to result in higher numbers of intrusions [40]. Finally, mechanisms of mnemonic control supporting direct suppression of episodic memory retrieval via hippocampal inhibition have been found in the right dorsolateral prefrontal cortex (DLPFC) [41]. Numerous previous works have tightly linked this region to executive cognitive control in tasks involving conflict [42–44], supporting the view that the DLPFC may also support emotional conflict adaptation [15].

The experimental index used in the present study approximates swiftly changing demands in individuals’ everyday life where participants frequently have to use implicit emotion regulation in response to multiple demands during their working day. The index estimates the susceptibility to trauma-related stimuli, which may signal potential threat and automatically trigger associated emotional and physiological responses resulting in intrusions. Over time, most people learn to discriminate between past trauma and trauma reminders in the present. In contrast, individuals who develop PTSD continue to relive their trauma memory as an experience in the "here and now" rather than an experience of the past [45, 46]. The cognitive control ability (in the case of PTSD, trauma cues) is likely to facilitate the process of learning to discriminate between threat posed by past trauma and by trauma memories. This possibility is supported by findings that resilient individuals flexibly and appropriately adjust attentional and emotional resources to meet the demands of a stressful situation [47] while individuals with generalized anxiety display reduced flexibility in regulating attention when facing unpleasant stimuli [48].

Our investigation contributes to the identification of specific mechanisms involved in the development and maintenance of intrusive emotional memories. We employed a prospective design that allowed us to distinguish between pre-existing memory mechanisms that may confer vulnerability to developing intrusive memories from those that are a consequence of such memories or symptoms of psychopathology [9]. If replicated in further studies, the finding that a deficit in attentional control contributes to the emergence of intrusive memories could inform prevention and intervention science. For instance, people at risk for trauma exposure (e.g., paramedics, soldiers in active duty, police officers etc.) could be specifically screened for cognitive control abilities. Those who score low on experimental tests of cognitive control may be referred to cognitive training as a preventative method to improve their ability to regulate attention in highly stressful situations [49, 50]. An additional method that might proof useful in the future is transcutaneous vagus nerve stimulation which has previously been shown to increase behavioral and electrophysiological markers of adaptation to conflict via arousal induction [51]. The enhancement of cognitive control by both training and neuro-stimulation may help to better cope with stressful situations and to reduce subsequent thoughts of these events.

Since there is no empirical evidence relating conflict measures from different modalities to intrusive memories we can only speculate on the generality of the underlying neural mechanism. Nevertheless, there have been numerous brain imaging studies investigating cognitive control and conflict adaptation using multiple types of tasks, such as for example the Simon-task, the Flanker tasks or the classic color-word-Stoop tasks, all inducing the need for cognitive control via response conflict [52]. A review of primate and human studies, along with a meta-analysis of the human functional neuroimaging literature, suggest that response errors, response conflict, and decision uncertainty elicits largely overlapping clusters of activation foci in an extensive part of the posterior medial frontal cortex (pMFC) [53]. Mechanistically, we believe that individuals with higher cognitive control may be better able to enhance task-relevant and decrease task-irrelevant neural representations during traumatic stimulation, i.e.: better shield their attention against distractors. In analogy, enhanced capability in cognitive control may also be beneficial in reducing traumatic cue interference in the aftermath of trauma during everyday life and thus reduce the occurrence of intrusive memories. Relatedly, a link between DLPFC and hippocampal memory inhibition has been previously reported, suggesting similar executive cognitive control mechanism also at work during active memory suppression [41].

The current study has several limitations. First, the current prospective relationship does not indicate a causal role for conflict adaptation in the prediction of intrusive memories. Future studies demonstrating causal effects would need to include specific experimental manipulations regarding cognitive control ability and observe subsequent effect on trauma-related symptoms. Future studies should consider overlapping constructs, including a non-emotional Stroop task. Together, the variables in our study explained 25% of the variance in intrusive memories, suggesting a significant association, but also involvement of other factors that influence the development of intrusions. Such factors may include a general responsivity of the noradrenergic arousal system. Specifically, heart rate, skin conductance and facial EMG reactivity during personal traumatic imagery was lower in an experimental group that had received post-trauma propranolol (a β-adrenergic antagonist that has been found to attenuate the consolidation of stressful memories in animal and humans) [54]. Factors that may contribute to the increased release of noradrenaline in response to sympathetic nervous system activation in PTSD include genetic or stress-induced decrements in neuropeptide Y (NPY) [55], which inhibits noradrenaline release, as well as a lower number or affinity of α2-adrenergic autoreceptors [56, 57]. Moreover, the level of cortisol has been well-recognized to have inhibitory effects on traumatic memory retrieval [58].

In addition, several brain regions have been associated with altered neural activity in PTSD patients as compared to controls and associated with intrusive memories. For instance, the hippocampus is involved in the encoding and recognition of episodic memories and environmental cues. As such hippocampal volume and activity may be related to trauma related emotional intrusive memories. Indeed, failure to recall extinction learning is associated with lower hippocampal activation in subjects with PTSD [59]. Moreover, individuals with PTSD exhibit greater insular cortex activation in response to fearful facial expressions, painful stimuli and memories [60, 61]. To clarify the unique contribution of cognitive control and identify additional factors contributing to intrusive memories in future studies, it would be helpful to include a parallel non-emotional task as well as adding other indices of cognitive control such as working memory capacity and resistance to proactive interference in future studies.

Notwithstanding its limitations, the current study has important methodological strengths and clinical relevance First, the current study is amongst the first to demonstrate a prospective relationship between pre-trauma cognitive control of emotional distraction and subsequent intrusive re-experiencing measured over one week using daily diaries. Second, unlike previous analogue research on the link between cognitive control and intrusive memories, we used emotional stimuli rather than non-emotional stimuli to measure individual differences in cognitive control. Third, the realistic trauma scene used here approximates the effects of witnessing or experiencing severe interpersonal trauma on intrusive memory. Fourth, our data show that for predicting intrusive memories, our behavioural marker of cognitive control outperformed a self-report measure of cognitive control ability. Most importantly however, because reaction time scores are behavioral manifestations of the underlying heterogeneity of task relevant neurobiological mechanisms, they can be used to study, quantify and predict pathology or even the risk to develop it in the future, as we did here. Taken together, our results suggest that the capacity to shield information processing from emotional distraction plays an important role in the development of intrusive emotional memories that merits further investigation. Our findings may inform prevention science and future clinical interventions aiming to reduce (cognitive) vulnerability to PTSD.
